# In Vitro Macrophage-Mediated Phagocytosis Assay of Brain Tumors

**DOI:** 10.7759/cureus.10964

**Published:** 2020-10-15

**Authors:** Madison Gardner, Jeffrey E Turner, Osama A Youssef, Samuel Cheshier

**Affiliations:** 1 Neurosurgery, University of Utah, Salt Lake City, USA; 2 Neurosurgery, Huntsman Cancer Institute, Salt Lake City, USA

**Keywords:** cd47, macrophages, phagocytosis, brain tumor, irradiation

## Abstract

Tumor-associated macrophages (TAMs) have recently emerged as potentially crucial therapeutic targets for cancer. Thus, the development of macrophage-mediated phagocytosis assays is vital for preclinical drug screening of different tumor cells. This assay can be used to evaluate the effect of anti-cancer therapy, such as immunotherapy, radiotherapy, and chemotherapy, on different tumor cells. Here, we describe the in-vitro phagocytosis assay in detail. As an example of immunotherapy treatment, we used a monoclonal antibody to block an anti-phagocytic signal (CD47) to evaluate the assay using human brain tumor cells and monocyte-derived macrophages. We also demonstrated that this assay can be used to evaluate the effect of different irradiation doses on the phagocytosis of brain tumor cells. This functional assay is fast, accurate, and highly reproducible. Furthermore, the results successfully demonstrate that anti-CD47 antibodies and irradiation can enhance the macrophage-mediated phagocytosis of brain tumors.

## Introduction

Macrophages are cells of the innate immune system present in almost all human tissues. Most macrophages are derived from monocytes in the peripheral blood circulation [1,2]. In solid tumors, the monocytes in the blood are recruited to the tumor microenvironment and differentiate into tumor-associated macrophages (TAMs) [3,4]. TAMs contribute to tumor development and progression by secreting different inflammatory cytokines [5]. In addition, tumor cells express specific surface proteins such as CD47, PD-L1, MHC-I, and CD24 that prevent macrophages from attacking them and may contribute to metastasis and tumor growth [6-8].

As a part of their function, macrophages perform an essential physiological process called phagocytosis. In this process, macrophages detect, engulf, and destroy foreign substances such as pathogens, apoptotic cells, and other abnormal invaders in the body. Phagocytosis of tumor cells is regulated by pro-phagocytic "eat me" and anti-phagocytic "don't eat me" signals through ligand-receptor interaction. Anti-cancer drugs that block "don't eat me" signals and/or increase "eat me" signals show promising potential in destroying tumor cells by macrophage-mediated phagocytosis [9].

Given the urgent need to develop a fast and accurate assay for anti-cancer drug screening, we have developed an in-vitro phagocytosis assay described here. This assay was successfully used to evaluate the effects of anti-cancer therapies, such as immunotherapy, radiotherapy, and chemotherapy, using human and mouse cells [10-15]. This method allows for a high diversity of treatments to be tested against different tumors.

Here, we describe the in-vitro phagocytosis assay using one of the human brain tumors and macrophages derived from human peripheral blood monocytes. Protocols for differentiating and labeling these macrophages are delineated in this assay. We use irradiation and anti-CD47 antibodies as an immunotherapy treatment to demonstrate the effectiveness of this assay in anti-cancer drug screening. Flow cytometry analysis was used to measure phagocytosis percentage. The development of this technique is essential in the preclinical development and study of novel macrophage-mediated therapies against tumor cells.

## Technical report

Reagents and equipment

Isolation of Peripheral Blood Monocytes from Donor Blood Filters

Falcon 70-µm Cell Strainers (Fisher Scientific, Cat #08-771-2)

Female Luer lock (Cole Parmer, Cat #EW-45501-04)

60-mL syringes

0.5 M EDTA, pH 8 (VWR, Cat #46-034-Cl)

Recovery Buffer: 5 µM EDTA pH 8 in DPBS

ACK Lysing Buffer (ThermoFisher, Cat #A1049201)

Ficoll-Paque Premium, 1.078 g/mL (GE Healthcare, Cat #17-5442-02)

Human CD14 microbeads (Miltenyi Biotec, Cat #130-050-201)

autoMACS® automated cell separator (Miltenyi Biotec, Cat #130-092-545)

Differentiation of Monocytes

Human M-CSF (ThermoFisher, Cat #34-8789-82)

Differentiation media: RPMI-1640, 10% FBS

In-Vitro Phagocytosis

96-well plate

RMPI-1640 (ThermoFisher, Cat #11875093)

DPBS (ThermoFisher, Cat #14190144)

CellTrace CFSE Kit (ThermoFisher, Cat #C34554)

Anti-CD47 antibody (Hu-5F9-G4) (FortySeven Inc.)

Irradiator (RS-2000)

Phagocytosis Measurement

Flow Cytometer (BD LSRFortessa™ Flow Cytometer)

DAPI (Thermofisher, Cat #D1306)

PE-CD11b antibody (BioLegend, Cat #101208)

APC/Cy7-CD14 antibody (BioLegend, Cat #325620)

Human Fc Block (BioLegend, Cat #564220)

Procedure

These methods delineate how to isolate and purify human peripheral blood monocytes from donor blood filters and differentiate them into healthy macrophage cells. The description details how to perform in-vitro macrophage-mediated phagocytosis of brain tumor cells and how to measure the phagocytosis percentage by flow cytometry. A humanized anti-CD47 antibody (Hu-5F9-G4) was used to enhance the macrophage-mediated phagocytosis efficiency.

Eluting and Isolating Human Peripheral Blood Monocytic Cells (PBMCs) from Donor Blood Filters

**Figure 1:**
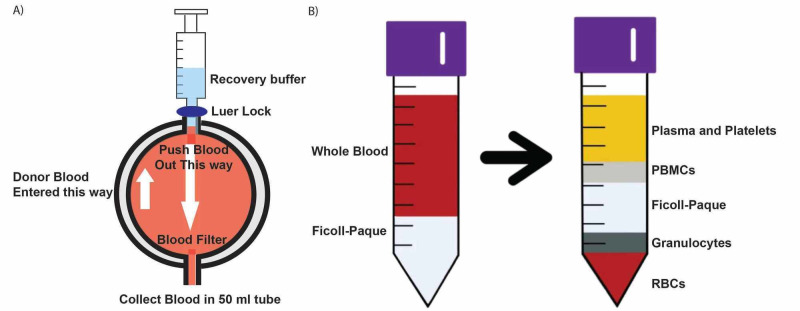
Isolation of peripheral blood monocytes (A) Elution of the whole blood from the donor blood filter. (B) Separation of PBMCs from the whole blood using Ficoll density gradient centrifugation.

The first step of this protocol includes isolating PBMCs from donor blood filters. Blood contains plasma, red blood cells, and white blood cells. In this step, we elute the blood from the blood filter, then isolate the buffy coat, and finally purify PBMCs from the buffy coat. There are a variety of blood filter types used by different blood banks. Here, we describe the elution of the buffy coat from specific blood filters (RC Leukocyte Filter For Blood, Haemonetics) (Figure 1A). However, this protocol can be used as a guide to elute the blood from different varieties of blood filters. These separated monocytes will be differentiated in later steps into macrophages.

a. Place an absorbent protective layer in the tissue culture hood to catch any drops of blood.

b. For each blood filter, prepare the following items.

○ One 50-mL conical tube to collect blood from leukofilters

○ Two 50-mL conical tubes containing 15-mL Ficoll-Paque Premium, 1.078 g/mL, warmed to 18°C to 20°C

○ One Luer lock adapter

○ 60-mL syringe

 

Eluting Leukocytes From Blood Filters

a. Place about 18 mL of Recovery Buffer into a 60-mL syringe and fill the rest of the syringe with air by pulling up on the plunger.

b. Clean a pair of scissors with 70% ethanol.

c. The blood must be flushed in the opposite direction that the blood was pushed through the filter at first. The arrow on the front of the filter indicates the direction the blood was initially pushed through the filter. Using the sterile scissors, cut the tube on the side where the arrow is pointing and snugly attach a Luer lock adapter (Figure 1A).

d. Connect the syringe onto the Luer lock adapter.

e. Open the 50-mL conical tube to catch the blood.

f. Cut the tube on the opposite side of the filter, maintaining the filter flat on the absorbent layer to avoid leaking blood.

g. Using the plunger on the syringe, forcefully flush the contents of the filter into the 50-ml conical tube.

h. Detach the syringe from the Luer lock, holding it to prevent leaking. Remove the plunger and reattach the syringe to the Luer lock.

i. Determine the total volume of liquid that was ejected from the filter (typically 30-40 mL). Fill the syringe with more Recovery Buffer to attain a final volume of approximately 50 mL, and repeat step No. 7. (Be sure to avoid too many bubbles. They should not be overflowing and escaping from the tube.)

j. Discard the filter and syringe into the biohazard waste.

k. Using the 25-mL pipette, remove the bloody bubbles at the top of the 50-mL tube and discard in the biohazard waste. Invert the 50-mL tubes to mix the blood solution gently.

Isolating PBMCs

a. Divide the 50 mL of blood solution equally into the two 50-mL tubes containing the 15 mL of Ficoll-Paque, being sure not to disturb the Ficoll-Paque layer at the bottom. To do so, tilt the tube almost horizontally and slowly pipette approximately 24-25 mL of blood solution. Repeat for the second tube. This tube will serve as a serum separator tube. The Ficoll layer must not be disturbed during this process; therefore, pipette the blood on top very carefully.

b. Centrifuge the tubes at 400 *g* for 30 to 40 minutes at 23°C with the brake turned off, ensuring the layers are not disturbed upon stopping.

c. After centrifugation, the red blood cells are now in the bottom Ficoll layer, the platelets and serum are on top, and PBMCs can be found in the middle buffy coat, as shown in Figure 1B. Using a 3-mL pipette, draw out the top two layers: the plasma and PBMC ring, being careful to leave the Ficoll-Paque layer alone. Combine the top two layers into a new 50-mL tube for each donor and discard the rest.

d. Pour in enough ice-cold PBS to each new tube to arrive at a total volume of 50 mL. Invert gently to mix. If there are aggregates or cell clumps, pass the solution through a 70-µm cell strainer into a new 50-mL conical tube. Centrifuge at 400 to 500 *g* for 10 minutes at 4°C to remove platelets and sediment leukocytes.

e. Aspirate the supernatant from the leukocyte pellet and discard.

Lysis of Red Blood Cells

a. Pipette 5 mL of ACK Lysing Buffer into each tube and pipette up and down approximately 10 times to resuspend the pellet completely. Place on ice for 4-5 minutes.

Note: If there are visible clumps while pipetting, strain using a 70-µm filter. Most blood filters require at least one straining during the process.

b. Within 5 minutes of adding the ACK Lysing Buffer, place enough cold PBS in the 50-mL tube to bring the volume to 50 mL. Centrifuge at 400 to 500 *g* for 5 minutes at 4°C.

c. Aspirate the supernatant.

d. Resuspend the pellet in 10 mL of RPMI/10% FBS media and count the cells.

e. Use the PBMCs immediately or freeze them in freeze media (90% fetal bovine serum (FBS), 10% dimethylsulfoxide (DMSO)).

Separation of Monocytes from PBMCs

This step is recommended for separating monocytes from lymphocytes in PBMCs. Human CD14 microbeads and autoMACS® automated cell separator were used to isolate monocytes from PBMCs according to the manufacturer's instructions.

3.0 Differentiation of Monocytes to Macrophages

This step describes how to differentiate monocytes into the macrophages needed to perform in-vitro phagocytosis.

a. Plate monocytes in differentiation media in the tissue-culture treated plate.

b. Add 25 ng/mL (final concentration) of human M-CSF.

d. Incubate the plate in a 37°C, 5% CO_2_ incubator for 5-7 days. Differentiated macrophages will adhere to the bottom of the plate. Check under a microscope and gently swirl the plate to see if cells are adhering. Adherent cells indicate successfully differentiated macrophages.

d. The macrophages can be maintained by removing the media and adding fresh differentiation media.

Dissociating Adherent Macrophages

This step explains how to dissociate adherent macrophages from the culture plate and wash off non-macrophage cells.

a. Aspirate media in the culture plate to remove non-adherent cells, and wash with 10 mL of DPBS twice.

b. Add 5-10 mL of TrypLE to the plate and incubate for 5-10 minutes at 37°C.

c. Verify that the cells are still attached by swirling the plate under a microscope. If the cells are still adherent, gently scrape plates with cell lifter to lift off adherent macrophages.

d. Transfer cells to a 50-mL tube, and add differentiation media to inhibit the TrypLE.

e. Spin the cells at 300*g* for 5 minutes at room temperature. Remove supernatant and resuspend cells in RPMI serum-free tumor media. Count viable cells using Trypan Blue and hemocytometer.

f. Keep on ice.

Labeling Tumor Cells with carboxyfluorescein (CFSE) Dye

This step explains how to label the tumor cells with a viable dye, such as CSFE. We use established brain tumor cells derived from surgery (all established brain tumor cells have been fingerprinted and authenticated at Stanford University). They form spheres in the stem cell media. It is worth mentioning that to achieve optimal phagocytosis of tumor cells by macrophages, we prefer to use patient-derived tumors in very low passages. Low passaged tumor cells better recapitulate the original tumor signature.

a. Collect the tumor cells in a 10-mL tube, and centrifuge at 300*g* for 5 minutes.

b. Carefully remove the media, and wash the cell pellet with DPBS.

c. Centrifuge at 300*g* for 5 minutes and remove the supernatant.

d. Add Accutase to the cell pellet and incubate at 37°C for 5 minutes. Count the viable cells using Trypan Blue and hemocytometer.

e. Use CFSE dye to label the tumor cells according to the manufacture's recommended protocol.

f. After washing the cells, resuspend cells in RPMI serum-free media. 

In-Vitro phagocytosis

Human brain tumor cells will be treated with anti-CD47 antibodies or different doses of irradiation prior to adding monocyte-derived macrophages. The ratio of macrophage to tumor cells is 1:2. In this protocol, we use 100,000 macrophages with 200,000 tumor cells. The final concentration of the anti-CD47 antibody is 20 ng/mL [13,14]. We irradiated tumor cells with 0, 2, 6, or 10 Gy three days before adding the macrophages.

a. In a 96-well plate, add 200,000 of CFSE-labeled tumor cells to each well. Add anti-CD47 antibody (+ CD47Ab), or IgG antibody (- CD47Ab, control) to each well and incubate for 30 min at 37°C to allow the antibody to block CD47 protein on the surface of the tumor cells. In the case of irradiation, tumor cells were irradiated with different irradiation doses and incubated for three days in the cell culture incubator (5% CO2, 37°C).

b. Add 100,000 macrophages to each well.

c. Incubate treated tumor cells with macrophages for at least 2 hours at 37°C.

4. Centrifuge the plate at 300*g* for 5 minutes at room temperature, and carefully remove the supernatant.

5. Add blocking buffer (95 µL of ice-cold 1% BSA-IgG free to 5 µL of Human TruStain FcX) and leave at room temperature for 5-10 minutes.

6. Add PE-CD11b and APC/Cy7-CD14 conjugated fluorescent antibodies to each well at predetermined optimum concentrations and incubated on ice for 15-20 minutes in the dark.

7. Centrifuge plate at 300*g* for 5 minutes, carefully remove the supernatant and wash cells in 200 µL of ice-cold DPBS.

8. Centrifuge plate again at 300*g* for 5 minutes and carefully remove the supernatant.

9. Add 200 µL of 500 ng/mL DAPI in DPBS to each well.

 10. Perform flow cytometric analysis.

Results

Differentiation of Monocytes to Macrophages Efficiency

**Figure 2:**
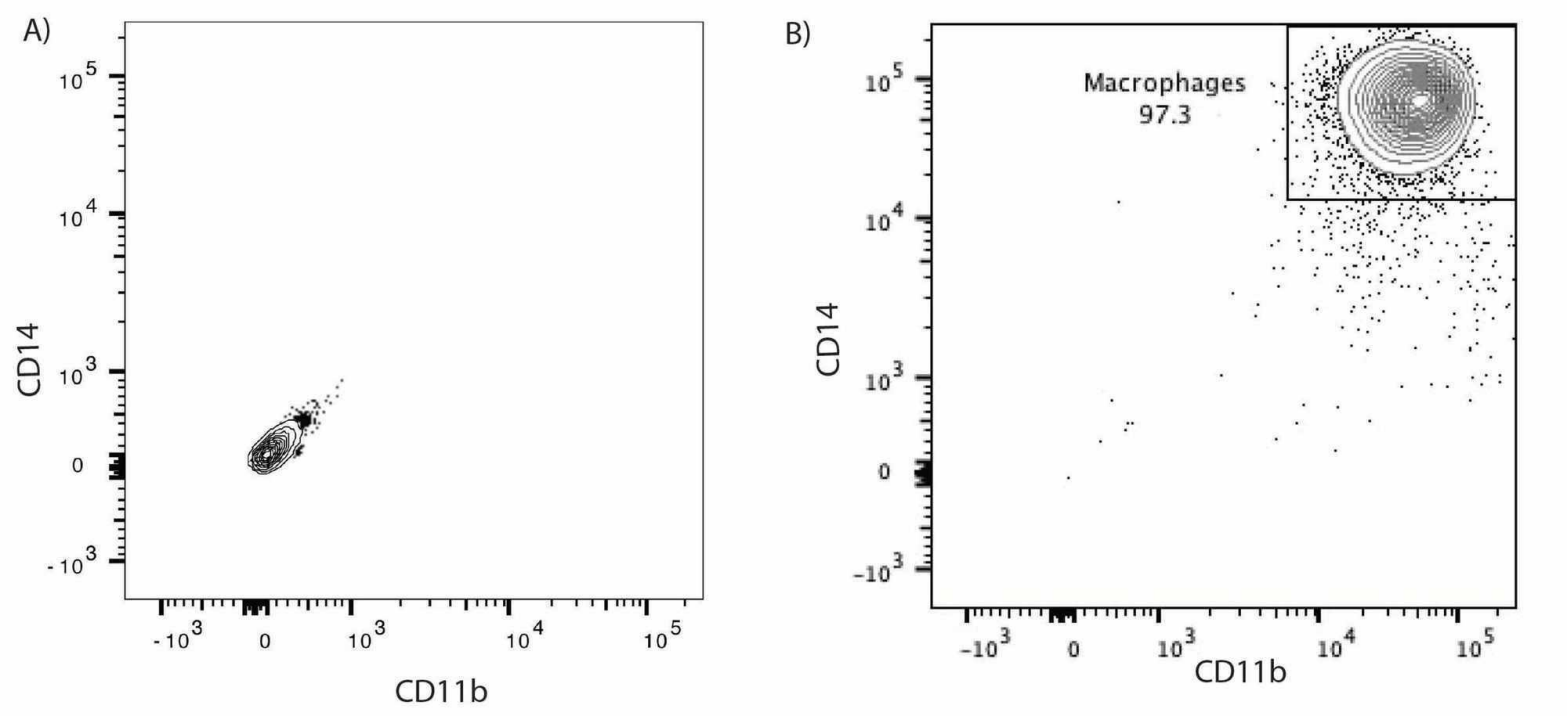
Flow cytometry analysis of human monocyte-differentiated macrophages (A) Unlabeled macrophages. (B) CD11b and CD14 fluorescently labeled macrophages.

The differentiation efficiency of monocytes to macrophages was evaluated by flow cytometry using fluorescently labeled CD14 and CD11b antibodies. As shown in Figure 2, about 97% of the live cells are macrophages (CD14-high, CD11b-high). These results demonstrate the effectiveness of our protocols in isolating, purifying, and differentiating PBMCs to macrophages.

In-Vitro Phagocytosis of Medulloblastoma

We use this protocol to perform in-vitro macrophage-mediated phagocytosis of one of the human pediatric brain tumors, medulloblastoma. We measured the percentage of macrophage-mediated phagocytosis using flow cytometry.

**Figure 3:**
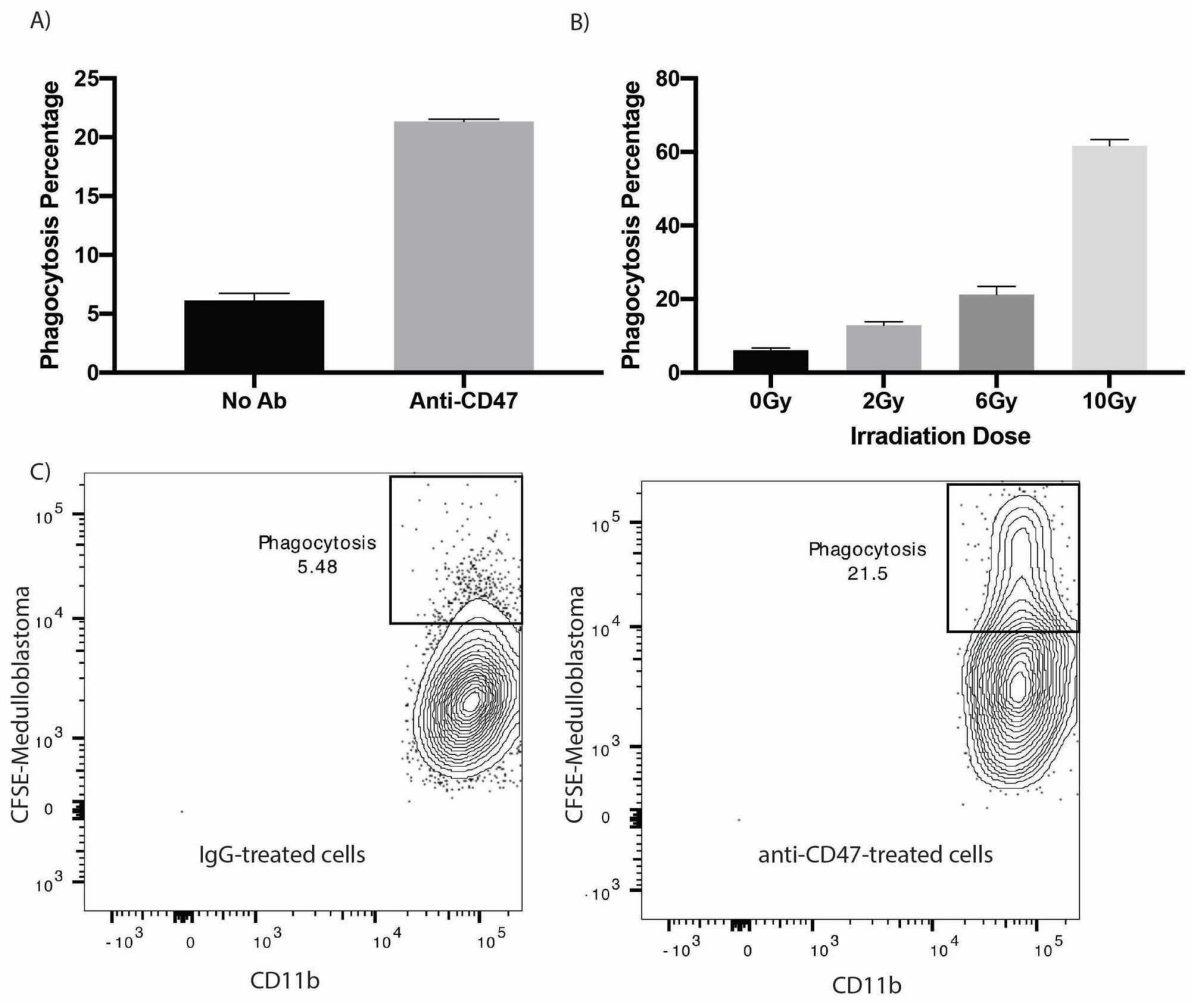
In vitro phagocytosis of medulloblastoma tumor cells (A) Percentage of macrophage-mediated phagocytosis of medulloblastoma ± anti-CD47 antibodies treatment (n = 3). (B) Percentage of macrophage-mediated phagocytosis of medulloblastoma after irradiating tumor cells with 0, 2, 6, and 10 Gy (n = 3). (C) Representative flow cytometry of phagocytosis assay of medulloblastoma without and with anti-CD47 treatment.

As shown in Figure 3A, C, approximately 5% of live tumor cells were engulfed by macrophages (CFSE-high CD11b-high). The addition of anti-CD47 antibodies to the tumor enhanced the phagocytosis to 21% (Figure 3A, C). These results show the application of our phagocytosis assay in evaluating possible immunotherapeutic treatment for brain tumors.

Figure 3B showed that irradiation enhanced the phagocytosis of medulloblastoma, and the phagocytosis efficiency increased with increasing the irradiation doses. This result is another demonstration of the effectiveness of our assay in evaluating the effect of radiotherapy on the phagocytosis of human brain tumors.

## Discussion

Macrophages are major components of the tumor microenvironment in many solid tumors. TAMs contribute to tumor development by increasing inflammation [11]. Moreover, tumor cells may escape immune surveillance by interacting with TAMs [6-8]. Therefore, macrophages represent promising targets for anti-cancer therapy in humans.

In the human immune system, the macrophage's primary function is to phagocytize pathogens and abnormal cells [16]. Phagocytosis is essential for maintaining normal homeostasis and healthy tissue, and it is a therapeutic target for a wide range of clinical applications [7-9]. Drug discovery is expensive and requires a large investment of resources and time. Consequently, there is an increasing demand for quick, sensitive, and accurate in vitro assay to screen multiple anti-cancer drugs.

We have developed an in vitro phagocytosis assay that is fast, accurate, and cost-effective. Unlike other phagocytosis assays, we use flow cytometry to measure the phagocytosis percentage. Flow cytometry enables a rapid, quantitative, and accurate measurement of phagocytosis efficiency. In this protocol, we used macrophages derived from human monocytes, but both macrophages derived from mouse bone marrow [12,13] and immortalized human monocytes such as THP-1 and U937 cell lines (unpublished data) have also been successful. Variability in human blood donors can affect the absolute phagocytosis percentage; however, the phagocytosis ratio of treated to untreated tumor cells is comparable from one donor to another. We previously showed that the in vitro phagocytosis assay displays different phagocytosis percentages with different primary brain tumor cells. The described protocol is specific to human brain tumors but may be successful for other tumor types, allowing greater generalizability for clinical treatments.

Immunotherapies that engage immune cells to fight against tumors prove to be powerful tools in combating cancer and are becoming increasingly utilized in clinical trials [6-9]. Here, we demonstrated the ability of our assay to evaluate the effect of blocking one of the "don't eat me" signals, CD47, on phagocytosis of human brain tumors. The results show that the anti-CD47 antibody and irradiation greatly enhanced the phagocytosis of medulloblastoma tumor cells (Figure 2B, C). These in vitro phagocytosis results were confirmed in animal models [13]. The developed platform has provided a great setting to interrogate the combined efficacy of other novel modalities with anti-CD47 antibodies, Particularly, It undergirds a basis for novel approaches against resistant tumors to conventional therapies or to anti-CD47 antibodies as a monotherapy [10-13].

The characteristic of drug resistance and the molecular and genomic heterogeneity of human tumors often pose substantial obstacles to effective cancer therapy. Therefore, combinations of anti-cancer treatments are likely to be required due to the heterogeneous nature of solid tumors. Our phagocytosis assay was successfully used to test different combinations of immunotherapy, radiotherapy, and chemotherapy [10-15].

## Conclusions

The establishment of an in-vitro phagocytosis assay provides a fast and reliable method to prescreen different anti-cancer drugs. It can be generalized to different tumor types as well as different anti-cancer drugs. This assay may reduce the unsuccessful screening of multiple drugs in animal models, in turn reducing the time and cost expended on animal models.
